# Platelet-platelet aggregates at single-event resolution as parameter in health monitoring

**DOI:** 10.1016/j.bbrep.2026.102555

**Published:** 2026-03-19

**Authors:** Sander Bekeschus, Lea Miebach, Broder Poschkamp, Sophie Tarnow, Linus Hübner, Julia van der Linde, Andreas Greinacher, Thomas Thiele, Jan Wesche

**Affiliations:** a Department of Dermatology, Venerology, and Allergology, Rostock University Medical Center, Strempelstr. 13, Rostock, 18057, Germany; b ZIK Plasmatis, Leibniz Institute for Plasma Science and Technology (INP), Felix-Hausdorff-Str. 2, Greifswald, 17489, Germany; c Department of Hematology and Oncology, Greifswald University Medical Center, Sauerbruchstr., Greifswald, 17475, Germany; d Department of Opthalmology, Greifswald University Medical Center, Sauerbruchstr., Greifswald, 17475, Germany; e Department of Transfusion Medicine, Greifswald University Medical Center, Sauerbruchstr., Greifswald, 17475, Germany; f Department of General, Visceral, Thoracic, and Vascular Surgery, Greifswald University Medical Center, Sauerbruchstr., Greifswald, 17475, Germany

**Keywords:** Diagnostics, Imaging flow cytometry, ISX, Neuronal network, Aggregates, Thromobocytes

## Abstract

Platelet analysis is crucial to assess in hemostasis, health, and disease. Current diagnostics primarily analyze bulk platelets, limiting the assessment of subtle, single-aggregate changes. We utilize Plateaggrate, a novel neuronal network-driven tool, for scoring platelet-platelet aggregation at single-event resolution using imaging flow cytometry (IFC) of labeled whole blood. This study evaluated Plateaggrate in 96 healthy human probands (61 males, 35 females; median age 39) to identify age and sex dependencies in native and ADP-stimulated platelets. Antibody-labeled platelets (CD42b and CD62P) were analyzed using IFC and segmented into singlets, doublets, triplets, and multiplets. While the weighted platelet aggregation score (WPA) showed no sex or age dependencies, Plateaggrate identified a slight but significant negative correlation of platelet singlets and total platelets with age. ADP-mediated activation, however, yielded robust platelet activation and aggregation independent of age, sex, and aggregate nature. In summary, this study validates the suitability of the WPA platelet algorithm for single-event platelet aggregation analysis in future disease patient cohorts.

## Highlights

• Plateaggrate, a neural network designed for analyzing single-cell platelet aggregation using imaging flow cytometry (IFC), was utilized to study platelet behavior
• Key findings included a slight, yet significant, negative correlation between platelet singlets/total platelets and age
• The weighted platelet aggregation (WPA) algorithm is validated for future use in analyzing single-cell platelet aggregation in disease patient cohorts

## 1 Introduction

In response to vascular injury, platelets adhere to the damaged endothelium, become activated, and aggregate in a process called hemostasis. This primary response is tightly linked to the coagulation cascade, which stabilizes the forming plug further through fibrin deposition [1]. Disruptions in platelet function or number can lead to a wide range of pathological conditions, including bleeding disorders and thrombotic diseases [2]. Beyond these, platelets have also been implicated in cancer progression, inflammation, and immune modulation [3,4].

Given their critical role in both physiological and pathological processes, accurately assessing platelet function at single-aggregate resolution appears plausible to employ in clinical diagnostics, but has not come into fruition so far due to the lack of respective measurement devices, as well as laboratory and software standardization. However, to measure platelet function in the bulk, a range of methods exists, which can be broadly categorized as being based on analysis by aggregation-inducing agents, shear stress, or flow cytometry [5]. Despite their utility, many of the methods rely on pharmacological agonists to activate platelets and can suffer from limitations related to accessibility, standardization, as well as the complexity of measurement and interpretation [6,7].

**Fig. 1:**
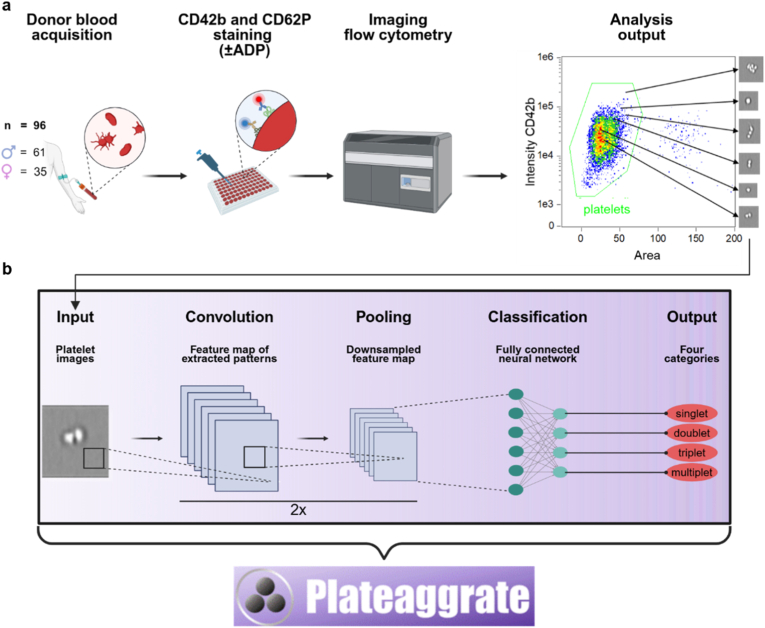
**Overview of experimental procedure and image processing by a convolutional neural network (CNN).** (**a**) Blood from 96 donors (61 males and 35 females) was collected in citrate tubes and transferred to 96-well plates. Samples were incubated with antibodies against CD42b and CD62P, with and without ADP stimulation. All samples were analyzed by imaging flow cytometry. Gating was performed to exclude non-platelet events from analysis, and platelet distribution with representative images of the platelet types is depicted. (**b**) Simplified overview of the neuronal network structure and image processing. Platelet images were used as input and subsequently convoluted and pooled two times. The output of the last convolution step was flattened in order to function as input for the fully connected NN, which assigns the images into one of four possible categories as its final output.

Moreover, methods are lacking to characterize more subtle platelet changes that may not necessarily result in overt aggregation but could still indicate an altered bleeding or thrombotic potential. Such an approach may be particularly valuable in assessing the risk of bleeding and thrombotic events pre- or postoperatively, monitoring of platelet function in cardiovascular disease, where platelets are significant targets for drug therapy, or evaluating the effects of therapeutic interventions in patients with rare bleeding and thrombotic disorders [8]. To this end, we have recently created a novel convolutional neural network-based tool for platelet analysis, called Plateaggrate. By utilizing single-event data obtained through imaging flow cytometry, Plateaggrate allows the scoring of platelet-platelet-aggregation in antibody-labeled whole blood samples (Fig. 1b), independently of platelet activation by agonists such as adenosine diphosphate (ADP) or collagen. A validation of the software and its implemented convolutional neural network (CNN) was previously conducted and demonstrated reliable performance in characterizing platelet aggregation [9]. Here, we aimed to evaluate Plateaggrate in a cohort of 96 healthy human probands to investigate potential age- and sex-related dependencies at baseline in terms of platelet-platelet doublets, triplets, and multiplets under native conditions and following ADP stimulation. We could successfully demonstrate the suitability of our method to characterize platelets at single-aggregate resolution.

## 2 Materials and methods

### 2.1 Blood collection

A total of 96 blood samples were obtained from 61 male and 35 female voluntary donors (median age 39) at Greifswald University Medical Center (Germany). Participants were eligible for blood donation upon providing informed consent and in the absence of any contraindicated underlying conditions or the use of relevant pharmacological drugs. To this end, all donors were considered generally healthy within the context of this study. Blood was collected using sodium-citrate (0.129 M, Vacutainer; BD Biosciences, Germany) as an anticoagulant. This study was approved by the local ethics committee (approval number: BB166/17) and conducted in accordance with the principles outlined in the Declaration of Helsinki.

### 2.2 Antibody staining and platelet activation by ADP

After collecting donor blood, experimental procedures were followed within 2 h after blood draw in batches of up to 12 samples. For the staining process, 5 μL of whole blood was incubated in technical duplicates for each sample with 0.6 μl CD42b antibody (conjugated to phycoerythrin-cyanine 7 (clone: HIP1; 0.48 μg/100 μL); BioLegend, The Netherlands) and 0.1 μl CD62P antibody (conjugated to allophycocyanine (clone: AK4; 0.20 μg/100 μL); BioLegend, The Netherlands) in 20 μL of phosphate-buffered saline (PBS) solution (Fig. 1a). PBS was used to maintain isotonic conditions while avoiding additional buffering components or divalent cations that could alter platelet reactivity. All samples were processed using identical buffer conditions to ensure comparability. For each sample, a baseline sample and an activation (positive) control were included. Baseline samples were antibody-stained in PBS, while activation controls were antibody-stained in PBS in the presence of the potent platelet-activator adenosine diphosphate (ADP, 0.1 mM; Sigma-Aldrich, Germany). After 15 min incubation at room temperature in the dark, samples were fixed by adding 225 μl of 0.5% paraformaldehyde (PFA; Sigma-Aldrich, Germany). The time between blood collection and fixation was strictly controlled and limited to a maximum of 2 h to minimize methodological variability. Due to practical constraints associated with donor blood collection and batch-wise processing, all samples were handled according to a standardized protocol, and baseline and ADP-stimulated samples from the same donor were processed in parallel. Measurements obtained within this time window showed minimal variation, indicating sufficient temporal stability for reliable platelet analysis and minimizing background platelet activation during sequential flow cytometry acquisition.

### 2.3 Imaging flow cytometry

In Imaging flow cytometry, cells in suspension are aligned into a single stream and passed through a flow chamber. Light transmission is recorded spatially resolved by a camera system, as opposed to traditional flow cytometers using photomultiplier tubes or avalanche photodiodes to record and integrate light emission per object identified. The ImageStreamX Mark II system (Amnis, Canada) used in this study features a co-linear laser setup, where each cell is exposed sequentially to three lasers emitting at wavelengths of 405 nm, 488 nm, and 642 nm, allowing the detection of multiple fluorescent markers. 10 × 10^3^ platelet complexes were recorded per measurement using conditional gating on camera-focused CD42 PE/CY7 events at a 1.0 μL/min flow rate and a blue laser power of 200 mW (Fig. S1). The conditional gating was key to the measurement, as this allowed us to keep file sizes for each measurement reasonable, as 99% of the objects in whole blood (mainly erythrocytes) were outside the CD42 gate and hence not stored in each file. Platelet numbers reported in figures refer to the total number of platelet events acquired and analyzed per measurement by imaging flow cytometry, rather than the absolute physiological platelet concentration in whole blood. For each acquisition, 20 μL of diluted whole blood was aspirated by the autosampler, and the volume required to acquire at least 3000 single platelets run through the ImageStreamX system was analyzed. A 642–745 nm bandpass filter was used for the brightfield channel, a 560–595 nm filter for CD42 b PE/Cy7, and a 435–505 nm filter for CD62P BV421 fluorescence emission capture. Data acquisition was performed using the INSPIRE software, and subsequent analysis was carried out with the IDEAS software (both Amnis, Canada) and the self-written open-source software Plateaggregate [9].

### 2.4 Image processing

The image data generated by imaging flow cytometry were imported into Plateaggrate software for automated analysis (Fig. 1b). Plateaggrate employs a convolutional neural network (CNN) to classify each image based on platelet aggregation patterns. Initially, the input images undergo two consecutive rounds of convolution and pooling operations, allowing the network to extract and condense relevant spatial features. The resulting feature maps from the final convolutional layer are then flattened and used as input for the fully connected layers of the neural network. As the final output, the CNN assigns each image to one of four predefined categories, representing different degrees of platelet aggregation (singlets, doublets, triplets, multiple platelet aggregates). More detailed descriptions of the structure of the program and its testing can be found in the previous report and its associated supplemental figures and software code provided at GitHub [9].

### 2.5 Weighted platelet aggregation

**Fig. 2:**
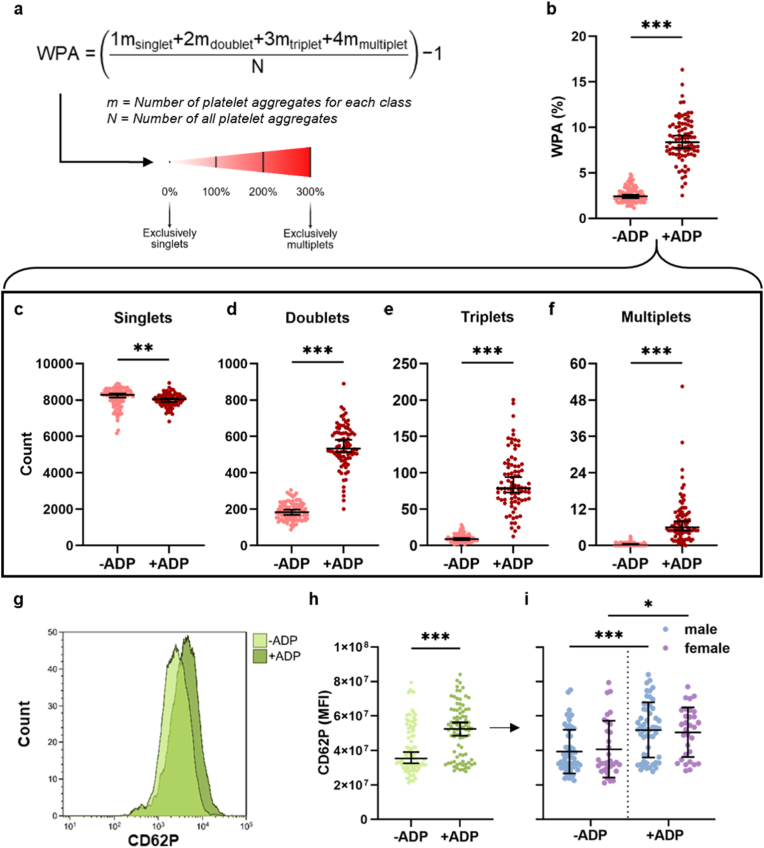
**Aggregation status and CD62P expression with and without ADP activation.** (**a**) Equation for weighted platelet aggregation (WPA); (**b**) WPA with and without ADP stimulation of whole blood; (**c-f**), subgroup quantification of (**c**) singlets, (**d**) doublets, (**e**) triplets, and (**f**) multiplets; (**g-h**) platelet activation indicator CD62P mean fluorescence intensity (MFI) of with and without ADP stimulation as representative flow cytometry histogram (**g**) and quantified across all blood donors (**h**); (**i**) CD62P MFI separated by sex. Statistical analysis was performed using Welch's *t*-test or Mann-Whitney test (*p < 0.05, **p < 0.01, ***p < 0.001; N = 80: m = 49; f = 31).

The previously introduced parameter [9] weighted platelet aggregation (WPA) was used to characterize the level of platelet aggregation further. It contextualizes the number *m* of recorded platelet aggregates in each class (e.g., singlets, doublets, triplets, multiplets) relative to the total amount of platelet complexes *N*. Its value ranges between 0% and 300%, where 0% represents exclusively singlets (no aggregation) and 300% corresponds to exclusively multiple platelet aggregates (multiplets). WPA was calculated by weighting each event class by the number of platelets it contains, summing these values, dividing by the total number of platelet events acquired, and subtracting the singlet baseline, according to the formula shown (Fig. 2a). Subtraction of baseline (1) normalizes the metric such that a sample consisting exclusively of singlets yields a WPA value of 0. The resulting WPA value is expressed as a percentage and represents the proportion of platelets participating in platelet-platelet aggregates.

### 2.6 Prefiltering of data

For each donor and sample (baseline vs. ADP-activated), measurements were performed in technical duplicates. This was done to quality control our method as we obtained duplicate measurements for all 96 donors, duplicate measurements for baseline and ADP-activation, leading to a reasonable high number of 192 conditions tested twice (two technical replicates) on the image cytometer and facilitating analysis of reproducibility. Note that the technical replicates were measured directly together (adjacently). To ensure that measurements did not deviate excessively for each blood sample, we defined an acceptable range of variability for the key Plateaggrate output, the WPA parameter. Based on data observations, the range was set between 70% and 130% of the mean value of the two technical replicates, which encompasses over 90% of replicate values. Measurements falling outside this range were considered outliers, and the corresponding donor data were excluded from further analysis. Using this criterion, seven of the 96 donors (approx. 7%) were omitted. Following this initial filtering step, an additional quality control measure was conducted. To ensure consistency across experimental days, the mean WPA values for each acquisition date were subjected to the "ROUT" outlier test (Q = 1%) integrated in GraphPad Prism (Fig. S2). Based on this analysis, data from nine additional donors were excluded.

### 2.7 Light transmission aggregometry

Light transmission aggregometry (LTA) was performed as described before [10], using collagen as a functional test to confirm platelet competence in a subset of donors. 180 μL platelet-rich plasma of the blood of each donor was used and incubated with 20 μL collagen (8 μg/mL; Takeda Pharmaceutical, Japan). Platelet aggregation was monitored at discrete time intervals of 5, 10, and 15 min using a 4-channel aggregometer (LABiTec, Germany). While continuous percent aggregation or slope was not calculated, the presence or absence of aggregation at these time points was recorded, confirming physiological platelet function and apparent absence of antiplatelet medications in the tested donors.

### 2.8 Statistical analysis

Formatting of raw data was performed in Excel (Microsoft, USA) and visualized using prism (GraphPad Software, USA). Statistical analyses were performed using Welch's *t*-test or Mann-Whitney test, or Pearson linear regression, where appropriate. Data is depicted as mean with standard deviation (SD) for normally distributed data and as median with 95% confidence interval for non-normally distributed data. Statistical significance is indicated as follows: *p < 0.05, **p < 0.01, ***p < 0.001.

## 3 Results

### 3.1 The WPA consistently identified platelet-platelet-aggregates

With the recently developed platelet-platelet-aggregation analysis tool Plateaggrate, we here aimed to analyze its robustness and reproducibility by characterizing the platelet aggregation levels in healthy human donor blood. Blood samples from 96 donors were collected and stained for the platelet surface marker CD42b and the platelet activation marker CD62P, both under native conditions (untreated blood) and with the addition of ADP to induce platelet activation. Following imaging flow cytometry, images of platelets were exported to the Plateaggrate software (Fig. 1a). Using a convolutional neural network architecture, Plateaggrate classifies input platelet images into four categories: singlets, doublets, triplets, and multiplets (Fig. 1b). Reasonable data size handling was achieved by conditional gating on CD42b^+^ and in-focus platelets (Fig. S1), reducing the data load by 99%. Based on the four Plateaggrate categories (platelets as singlets, duplicates, triplets, and multiplets), the previously established [9] weighted platelet aggregation (WPA) score was calculated (Fig. 2a) for each donor sample under native and ADP-activated conditions. Non-activated samples showed a baseline WPA of approximately 2.5% with a standard deviation of ±0.8% that increased on average to about 9% upon ADP stimulation (Fig. 2b). Since all donors were prefiltered against apparent disease and medication use, and thus can be considered generally healthy, this baseline score of 2.5% seemed to reflect a normal, regular baseline aggregation score in our study cohort. In numbers, in about 10,000 recorded platelets – after exclusion of images with platelets and erythrocytes – on average, approximately 8400 were identified as singlets (Figs. 2c), 200 as duplicates (Figs. 2d), 10 as triplets (Figs. 2e), and 0-1 as multiplets (Fig. 2f). This changed dramatically upon the addition of ADP were stimulation increased the mean WPA score 3.3-fold, driven by a marked rise in doublets (approx. 500, a 2.5-fold increase), triplets (approx. 70, a 7-fold increase), and multiplets (approx. 5, a 5-fold increase), and accompanied by a corresponding significant decrease in singlets (Fig. 2c–f). CD62P analysis further confirmed platelet activation by ADP (Fig. 2g), as indicated by a shift toward higher fluorescence intensities (Fig. 2h). Importantly, this activation was observed equally in both male and female donors, demonstrating that platelet responsiveness to ADP was independent of sex, while not showing any significant differences between sexes (*p* = 0.70) (Fig. 2i).

### 3.2 Baseline WPA shows a modest age association in male, but not female, healthy donors

**Fig. 3:**
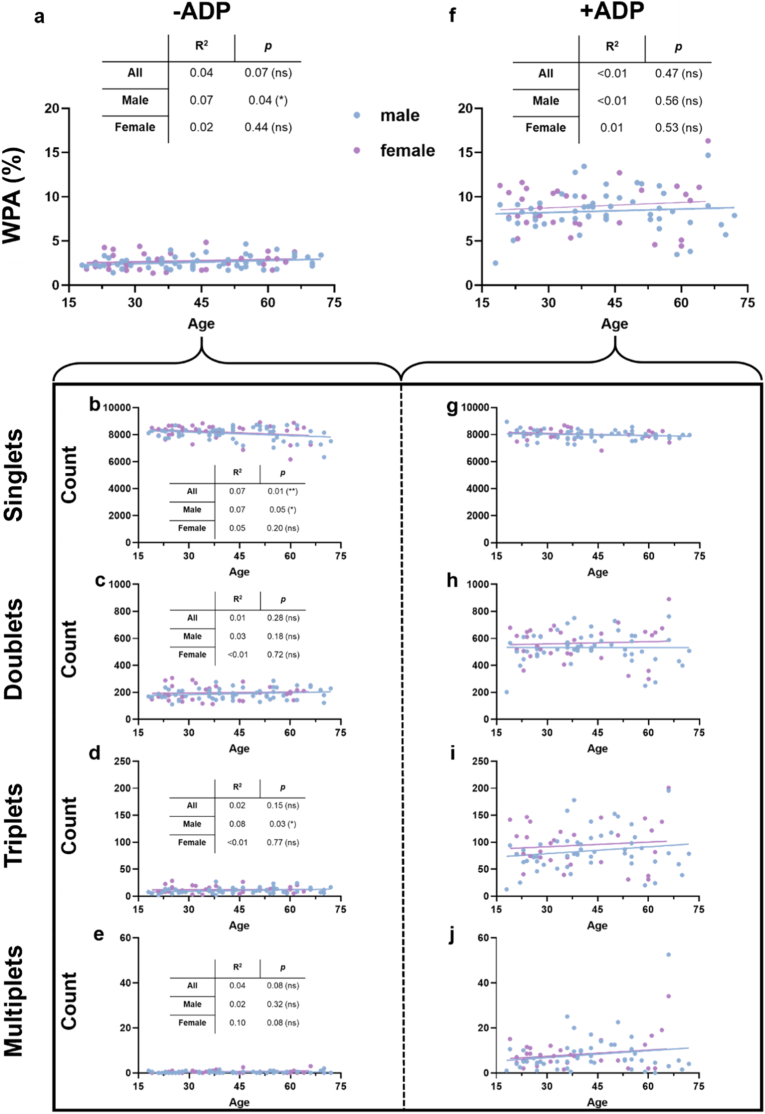
**Weighted platelet aggregation (WPA) stratified for sex and age.** (**a-e**) WPA (**a**) without ADP application and subgrouped by number of singlets (**b**)**,** doublets (**c**), triplets (**d**), and multiplets (**e**); (**f-j**) WPA (**f**) with platelet activation by ADP subgrouped by number of singlets (**g**), doublets (**h**), triplets (**i**), and multiplets (**j**). Statistical analysis was performed using linear regression (*p < 0.05, **p < 0.01, ***p < 0.001; ns = non-significant; N = 80: m = 49; f = 31).

We next assessed whether there was a correlation between the WPA score and age. In the absence of ADP activation (i.e., native donor blood), a statistically significant but modest positive correlation of baseline WPA with age was observed in male donors, whereas no correlation was seen in female or combined donor groups (Fig. 3a). The low R^2^ values indicate that age explains only a small fraction of the variability in WPA, suggesting that other biological or environmental factors also contribute to inter-individual differences. When resolving these data for the platelet aggregation categories, a weak negative correlation was found for singlet platelets and all and male but not female donors (Fig. 3b). For duplicates, no significant age correlation was found (Fig. 3c), while a modest but significantly positive correlation was found for platelet triplets in males but not females or all blood donors (Fig. 3d), suggesting that triplets are a key contributor to the observed WPA trend. For multiplet platelet aggregates, no significant association with age was found (Fig. 3e). The results showed no trends with age when human donor blood was spiked with ADP for platelet activation (Fig. 3f). Technically, it should be noted that ADP activation did not interfere with the ability of unambiguously identifying platelets by imaging cytometry, as similar platelet counts (Fig. S3a) and similar mean fluorescence intensities of the platelet marker CD42b used for gating (Fig. S3b) between native and ADP-stimulated whole donor blood suggested. Generally, ADP-stimulated platelets showed no significant correlation with age for singlets (Fig. 3g), doublets (Fig. 3h), triplets (Fig. 3i), or multiplets (Fig. 3j). In general, these measurements did not reveal any differences between sexes. Specifically, WPA scores (Fig. S4a) and platelet counts (Fig. S4b) were similar between both sexes, independent of comparison within native and ADP-stimulated whole blood data on platelets. The same findings were made for subpopulation analysis of platelet aggregates in both sexes for singlets (Fig. S4c), doublets (Fig. S4d), triplets (Fig. S4e), and multiplets (Fig. S4f). Upon correlating the general WPA score with the CD42b intensities, no significant change was observed in native blood samples (Fig. S5a). However, a significant positive correlation was identified in stimulated samples, indicating an increase in CD42b expression from the addition of ADP (Fig. S5b). Interestingly, a significant negative correlation was found between the WPA score and CD62P expression in native blood (Fig. S5c), while a positive correlation was observed for ADP-stimulated platelets (Fig. S5d). This difference may reflect partial masking of CD62P epitopes in native blood, arising from structural differences between non-activated and activated platelets. In small basal aggregates, platelet-platelet contacts can sterically hinder antibody access to CD62P. Upon ADP stimulation, robust degranulation and membrane exposure of CD62P increase epitope availability, resulting in the observed positive correlation with WPA.

### 3.3 Data set validation age dependency platelet numbers

**Fig. 4:**
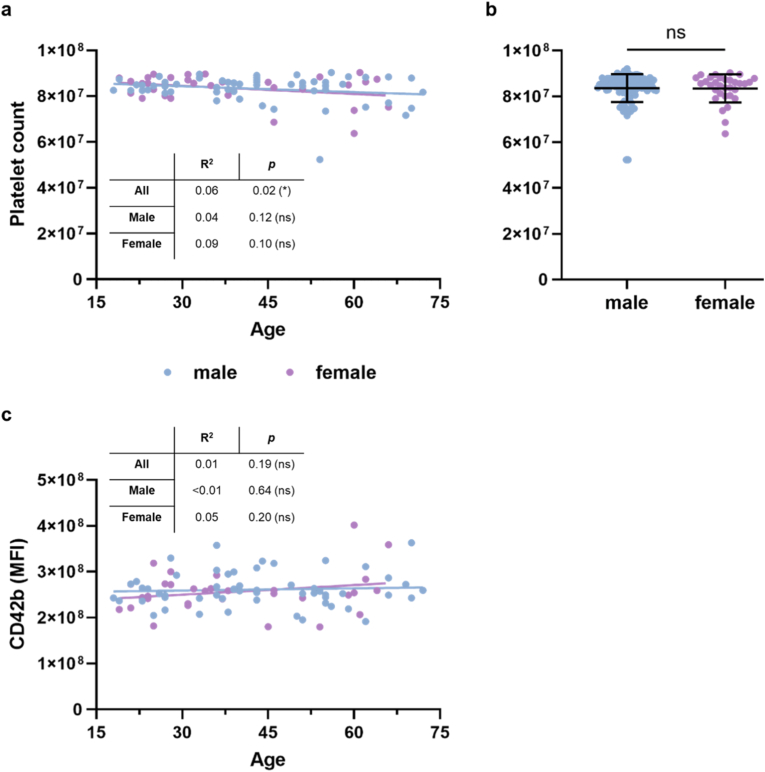
**Platelet count and CD42b expression without ADP activation stratified by sex and sex and age**. (**a-b**) Number of platelets decreased with age (**a**) and cumulated platelet numbers by sex (**b**) (platelet numbers indicate the total number of platelet events acquired and analyzed by imaging flow cytometry); (**c**) CD42b mean fluorescence intensity (MFI) was independent of age. Statistical analysis was performed using Mann-Whitney test (**b**) and linear regression (**a**, **c**) (*p < 0.05, **p < 0.01, ***p < 0.001; ns = non-significant; N = 80: m = 49; f = 31).

To complete the analysis of our study cohort, platelet counts were correlated against age, and a significant negative association was identified for platelet numbers and age (Fig. 4a). However, there were no differences between sexes (Fig. 4b). However, for ADP-stimulated platelets, a significant negative association was identified for platelet counts and age in both (but not each of the) sexes (Fig. S6a). To analyze whether our method could be potentially altered by differences in CD42b expression in dependence on age, both parameters were correlated, and no association was found in native donor blood for both and individual sexes (Fig. 4c). Similar findings were made for ADP-stimulated platelets (Fig. S6b). Finally, there was no age-dependent association of CD62P expression in native (Fig. S6c) and ADP-stimulated donor blood (Fig. S6d), independent of the sex analyzed. To verify that the donors of our cohort were healthy, the blood of approximately half of the participants was additionally tested for their platelets' competence to become activated using light transmission aggregometry. When a blood vessel is injured, the underlying collagen of the vessel wall is exposed. This exposure is the signal that causes platelets to aggregate, which can be measured in an aggregometer. Although LTA was performed only with collagen and not additionally with ADP, the strong increase in WPA following ADP stimulation demonstrates that our imaging flow cytometry-based assay is sensitive to agonist-induced platelet activation. All but one of the tested samples showed aggregation at 5 min post collagen addition (with one sample after 10 min) (Fig. S7a), indicating physiological platelet function and apparent absence of antiplatelet medications like aspirin in the tested blood donors. To verify that the selection of probands tested with collagen was representative in our study cohort, the tested probands’ blood WPA scores were plotted into the results of the entire study cohort, demonstrating no apparent deviation from scores obtained with non-tested whole blood donors (Fig. S7b).

## 4 Discussion

In this study, we demonstrate that our platelet-platelet aggregometry tool, based on single-event resolution using imaging flow cytometry, can accurately characterize platelet aggregates in native whole blood samples in a sizable cohort of blood donors. While a fraction of the detected micro-aggregates may arise during blood collection or early sample handling, the reproducibility of baseline WPA values across donors and the clear separation from ADP-induced aggregation suggest that this signal reflects a biologically meaningful baseline state measurable under controlled ex vivo conditions. Overall, we demonstrated the general applicability of this approach and our methodological protocols in characterizing platelet function in human donor blood, potentially opening up new research avenues in thrombosis and hemostasis, transfusion medicine, and cardiovascular disease medicine.

The utilization of imaging flow cytometry coupled with the Plateaggrate algorithm provides a distinct methodological advantage in assessing platelet function. Unlike traditional LTA, which yields a macroscopic measure of aggregation in platelet-rich plasma [11], this technique enables the granular, visual classification of aggregates (singlets, doublets, triplets, multiplets) within the more physiologically relevant environment of whole blood. The establishment of a WPA of approximately 2.5% in unstimulated, healthy donors provides an important reference range, indicating the presence of low-level platelet-platelet interactions detectable under standardized ex vivo conditions.

A limitation of the study was that the assay cannot definitively distinguish between micro-aggregates formed in vivo and those potentially generated during blood collection or preprocessing. To confirm preserved platelet competence within the donor cohort, collagen-induced LTA was performed in a subset of participants. All but one sample demonstrated aggregation within 5 min, indicating intact global platelet responsiveness. In this context, collagen-based LTA served as a general functional control to exclude overt platelet dysfunction rather than to validate P2Y12-specific signaling. Receptor-specific responsiveness is an important aspect of platelet biology, and preserved aggregation in response to one agonist does not formally exclude altered signaling through another pathway. ADP responsiveness was directly assessed within the WPA assay itself, where consistent and robust increases in aggregate formation were observed across donors, indicating intact P2Y12-mediated signaling. It should also be noted that, because LTA was performed solely as a qualitative functional check, detailed kinetic parameters were not systematically recorded. These metrics are important in conventional LTA for characterizing platelet responsiveness and aggregation dynamics, and their absence limits direct quantitative comparison with WPA measurements. Future studies designed to directly compare WPA with standard aggregation assays should incorporate full LTA parameterization, including lag time, maximal aggregation, and receptor-specific agonists, to enable comprehensive cross-method validation.

Importantly, although the absolute increase in WPA following ADP stimulation appears numerically modest, it represents a biologically meaningful, multi-fold enhancement of platelet-platelet interactions at the single-event level. Even under strong agonist stimulation, platelets do not uniformly form large aggregates. Instead, activation primarily increases the frequency and stability of small aggregates, such as doublets and triplets, while the majority of platelets remain as singlets. Consequently, relatively small absolute changes in WPA correspond to substantial shifts in platelet interaction dynamics. This highlights the sensitivity of the WPA metric to both basal and agonist-induced platelet activation, particularly in detecting subtle changes that are not captured by bulk aggregation assays relying on large-scale platelet clumping. Differences in the absolute abundance of multiplets compared with our earlier work add to that and likely reflect the larger cohort size, stricter prefiltering criteria, and absolute event-based reporting used in the present study. However, both datasets consistently demonstrate that higher-order platelet aggregates are rare under unstimulated conditions and increase robustly following agonist stimulation. Strict standardization of anticoagulation, processing, and fixation conditions minimizes the potential impact of ex vivo handling and allows reliable relative comparisons across samples. This approach is intrinsically more sensitive to subtle, subclinical changes in platelet activation than assays relying on agonist-induced, large-scale aggregation events. Moreover, circulating platelet aggregates are known to bind tumor cells and contribute to hematogenous metastasis [12], highlighting an additional potential application of this method.

Interestingly, we observed a negative correlation between WPA and CD62P expression in native, unstimulated samples, whereas a positive correlation was seen following ADP stimulation. This difference likely reflects partial masking of CD62P epitopes in small basal aggregates due to structural differences between non-activated and activated platelets. While antibody binding to CD62P could be sterically hindered in basal platelet aggregates, ADP stimulation induces robust degranulation and membrane exposure of CD62P, likely increasing epitope accessibility and resulting in a positive correlation with WPA. Importantly, the patterns of singlets, doublets, triplets, and multiplets were consistent across technical replicates, and samples were anticoagulated with citrate and carefully gated for CD42b positivity and focus quality. These considerations indicate that the measured aggregates represent bona fide platelet aggregation rather than nonspecific platelet agglutination. CD62P expression was quantified exclusively as MFI rather than percentage positivity. MFI captures both the fraction of expressing platelets and the extent of surface expression per platelet, making it a more sensitive and informative measure of activation, particularly in the context of aggregate formation, where epitope accessibility can vary. Additionally, our primary aim was not to define the total proportion of quiescent versus activated platelets at baseline, but rather to assess relative changes in activation following ADP stimulation. While percentage positivity could provide information on how sample handling influences basal activation, this was not the focus of our study. Using MFI thus allowed us to robustly capture shifts in platelet activation dynamics between unstimulated and ADP-stimulated states. Collectively, these findings demonstrate that our imaging flow cytometry-based assay reliably captures both basal and agonist-induced platelet activation while accounting for differences in receptor accessibility and aggregate structure.

A central observation of this study is a subtle, age-dependent increase in baseline platelet aggregation in males, whereas no such trend was apparent in females. While it is widely accepted that overall platelet reactivity increases with age due to factors like oxidative stress [13], our data support this concept by demonstrating that the age-related increase, while weak, was more pronounced in male over female donors. This sex-specific divergence suggests that underlying physiological mechanisms, potentially related to hormonal status, may modulate the age-dependent progression of basal platelet activation [14]. The continued hormonal milieu in pre- or peri-menopausal women may confer a protective effect. In contrast, oral contraceptives and smoking are known to have detrimental effects on platelet behavior in women [15], while the male response follows a more direct, cumulative trajectory of age-associated hyper-reactivity [16]. However, the lower number of women than men tested in our study could have also contributed to these findings and awaits further confirmation for higher-powered future reference measurements with our here described WPA score obtained by imaging flow cytometry. Additionally, other physiological or environmental factors likely contribute to baseline platelet behavior. Overall, our findings are generally in line with the literature, supporting the robustness of the WPA method for assessing platelet aggregation. However, at this moment, the present data do not justify age- or sex-specific reference values for WPA. The observed hyper-reactivity was previously suggested to relate to additional factors, such as platelet lipid membrane changes, e.g., higher cholesterol [17]. Hypercholesterolemia, in turn, positively associates with age, specifically in obese people [18], which may contribute to the age-related hyper-reactivity observed in our study. In addition, we found that the age-related WPA increase among males was driven predominantly by an increase in platelet triplet formation, rather than larger multiplets or smaller doublets. This detailed size classification provided by the Plateaggrate tool indicates an increased stability or formation rate of small, three-platelet aggregates, suggesting that men develop a pro-aggregatory platelet state while aging. Interestingly, others have found that especially platelets from females lose their ability to interact with von Willebrand factor when measuring platelet translocation on VWF, adding to a more prothrombotic capacity of platelets in older males [19]. Our findings may be the result of a rather persistent, low-level activation that pushes the equilibrium toward micro-aggregate formation, potentially making the circulating platelets hyper-responsive and 'primed' for subsequent activation in case of an injury, as previously suggested to be linked with elevated CD62P on platelets [20]. However, neither the male nor female cohort of our study showed an age-related positive association with CD62P, making the connection to the higher WPA less likely.

In our hands, the Plateaggrate tool is feasible to demonstrate platelet function differences at the single-event level. Depending on the experimental or clinical question, WPA can be assessed either in unstimulated whole blood to capture basal platelet micro-aggregation or in paired measurements before and after agonist stimulation to quantify platelet reactivity. Collectively, our work moves beyond the generalized concept of "increased platelet function" in the elderly [21], by providing a tangible, quantified metric that goes beyond previous flow cytometry assays on platelet aggregation [22]. While the present study is limited to a healthy reference cohort, the methodological framework and WPA score established here provide a robust baseline against which platelet aggregation patterns in disease states can be compared. Future application of this approach in patient cohorts with thrombotic, bleeding, inflammatory, or malignant disorders may allow the detection of subtle, disease-associated alterations in platelet behavior that are not captured by conventional bulk assays. Importantly, the WPA score quantifies the proportion of platelets engaged in micro-aggregates, a feature that has been associated with cardiovascular risk: previous studies have shown that circulating platelet aggregates, including platelet-leukocyte aggregates, are elevated in patients with cardiovascular disease and correlate with disease severity and clinical outcomes [[23], [24], [25]]. Therefore, WPA measured by the Plateaggrate platform may serve not only as a research tool but also as a potential biomarker for assessing platelet activation and stratifying cardiovascular risk.

## Author contributions

Conceptualization: S.B.; methodology: S.B., L.M., and B.P.; validation: L.M. and L.H.; formal analysis: L.M., B.P., S.T., and L.H.; investigation: L.M. and S.T.; data curation: L.M. and L.H.; visualization: L.M. and L.H.; supervision: S.B., L.M., B.P., J.v.d.L., A.G., T.T., and J.W.; funding acquisition: S.B.; writing – original draft: S.B., L.H., and J.v.d.L.; writing – review and editing: S.B., L.M., B.P., L.H., A.G., T.T., and J.W.

## Funding

This work was partially supported by the 10.13039/501100002347German Federal Ministry of Education and Research (BMBF/BMBTR, grant number 03Z22DN11 to S.B.).

## Declaration of competing interest

The authors declare that no conflict of interest existing with publication of this article.

## Data Availability

Data will be made available on request.
