# Supplementary figures and images for: Platelet-platelet aggregates at single-event resolution as parameter in health monitoring
Source: Biochem Biophys Rep. 2026 Mar 19;46:102555. doi: 10.1016/j.bbrep.2026.102555 (PMC13019091; doi:10.1016/j.bbrep.2026.102555)

## Figure S1


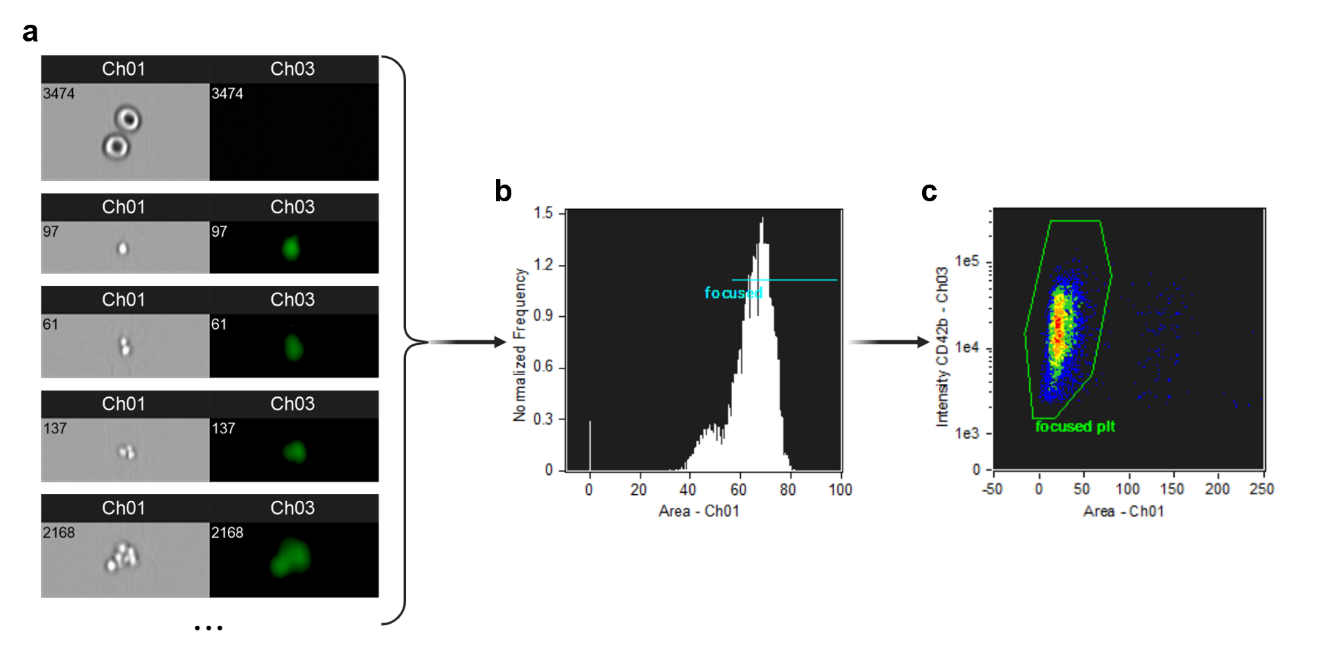


## Figure S2


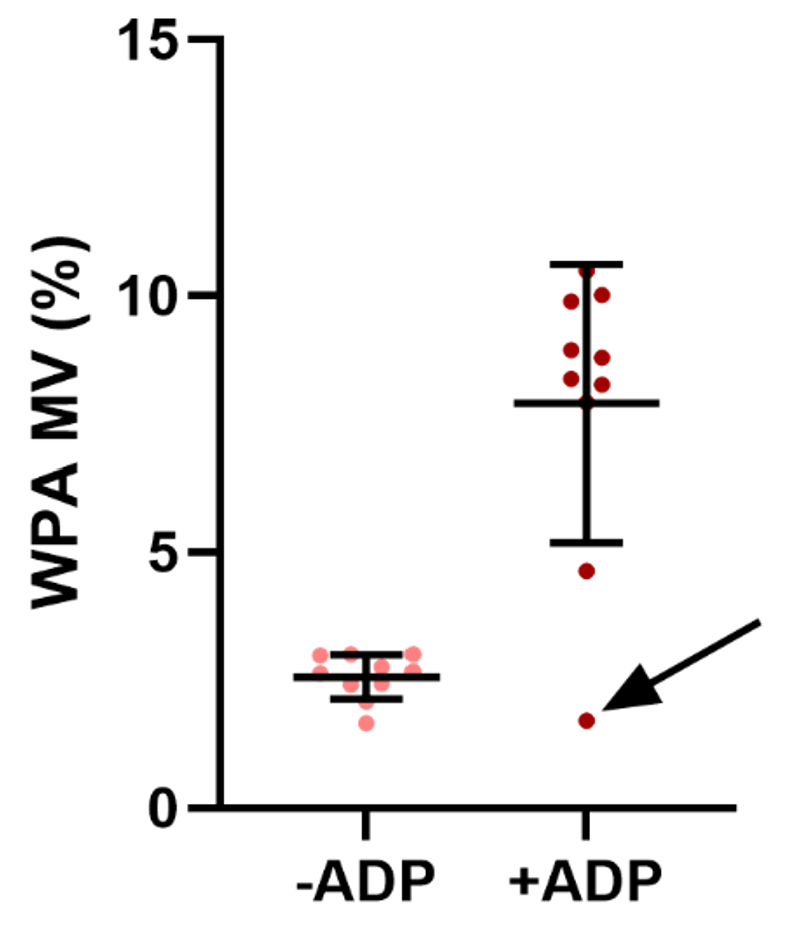


## Figure S3


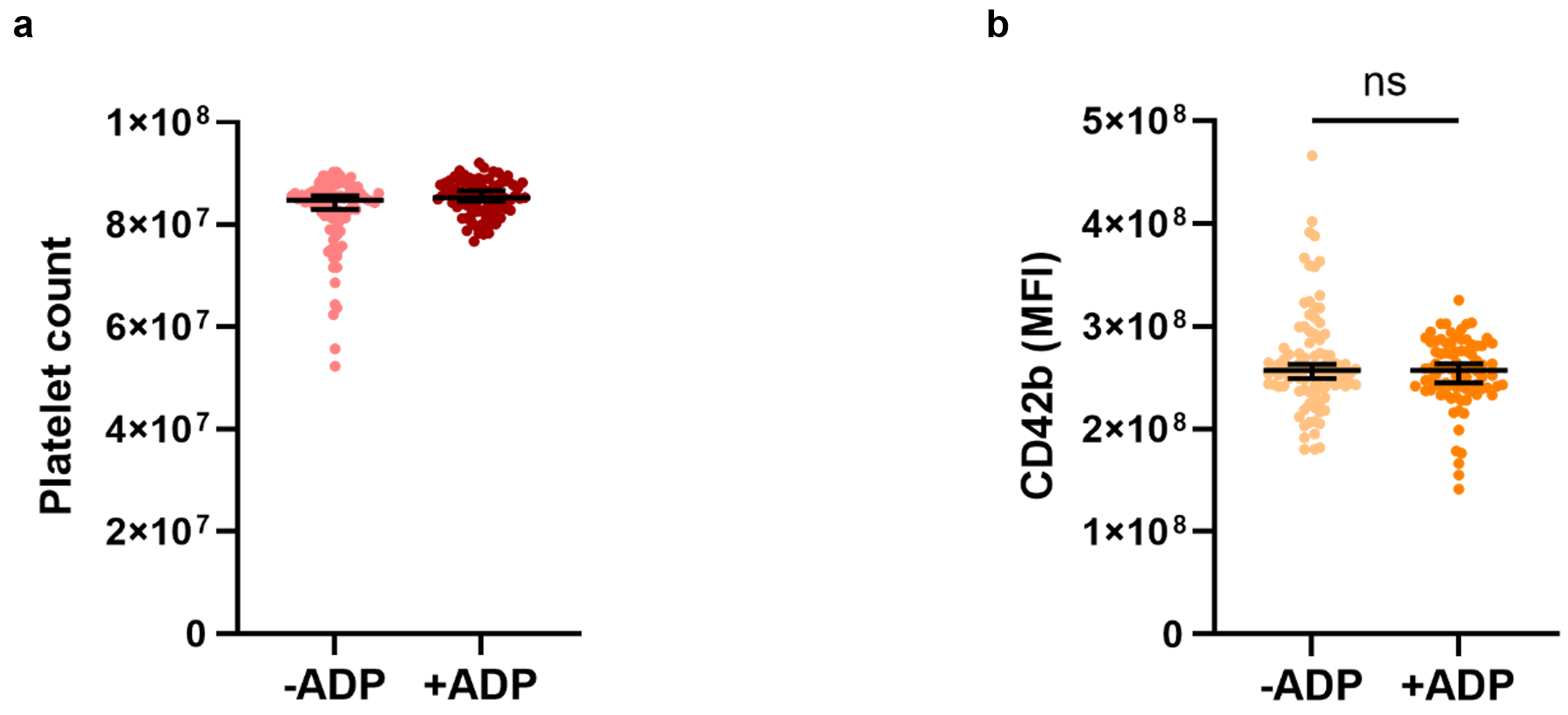


## Figure S4


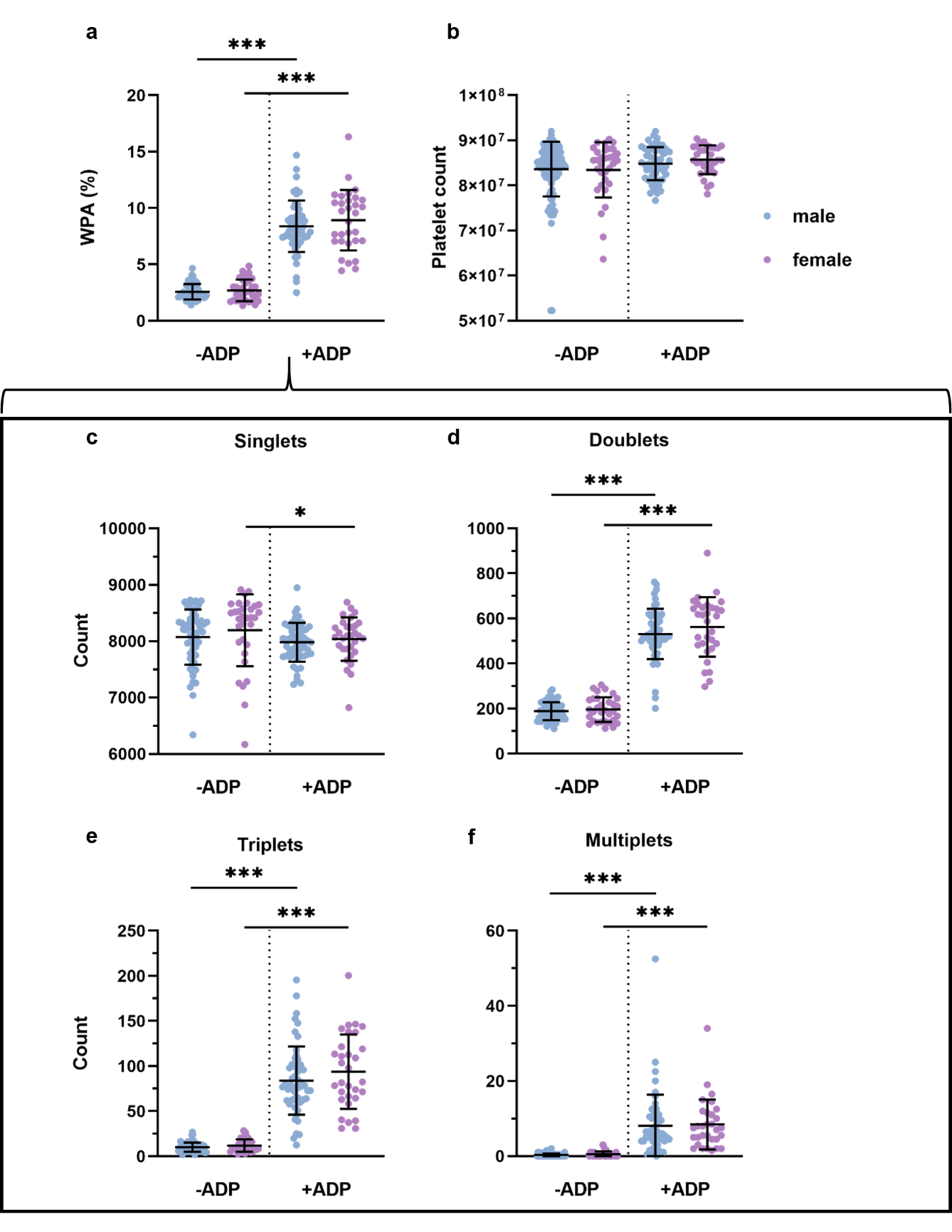


## Figure S5


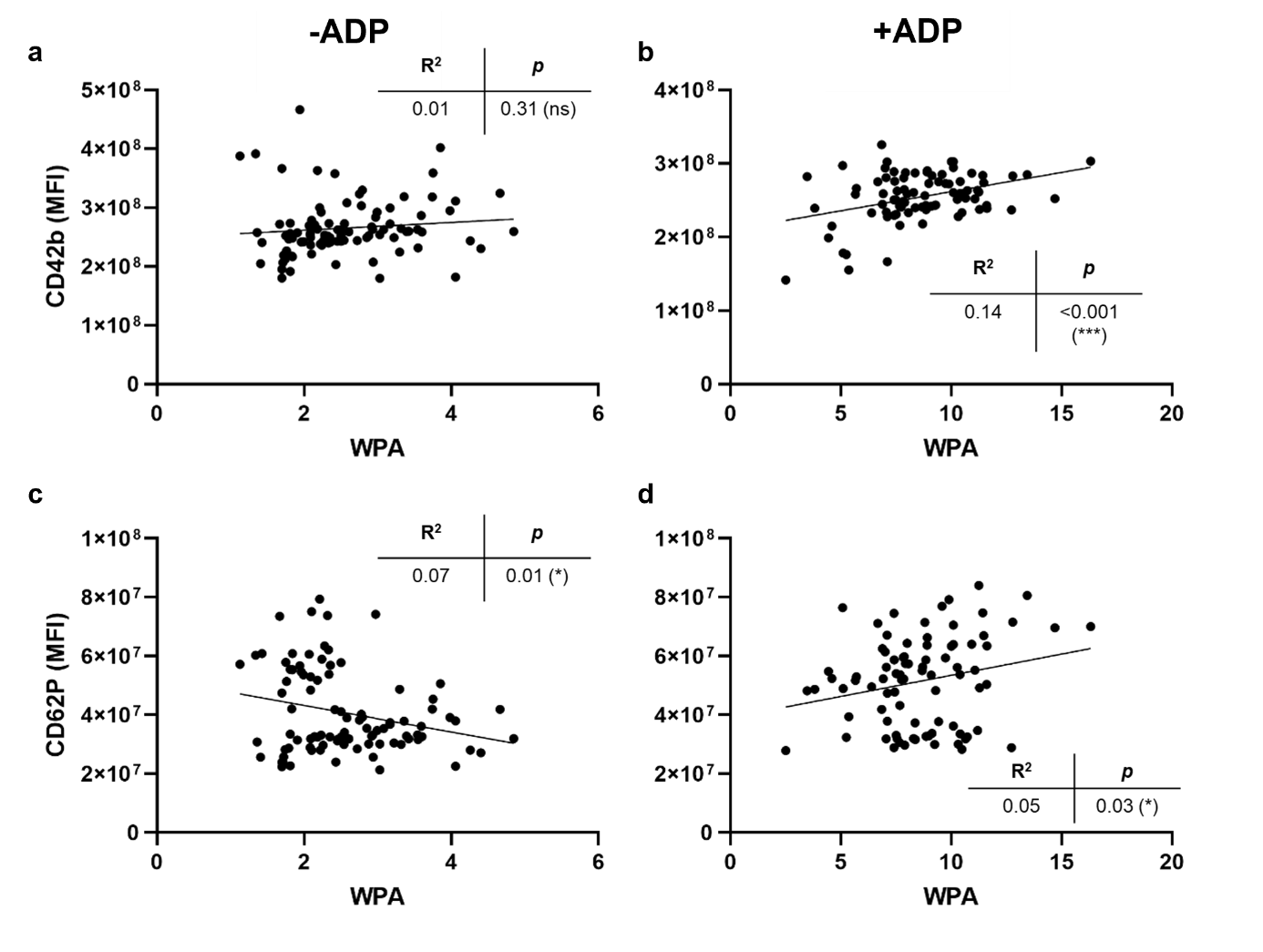


## Figure S6


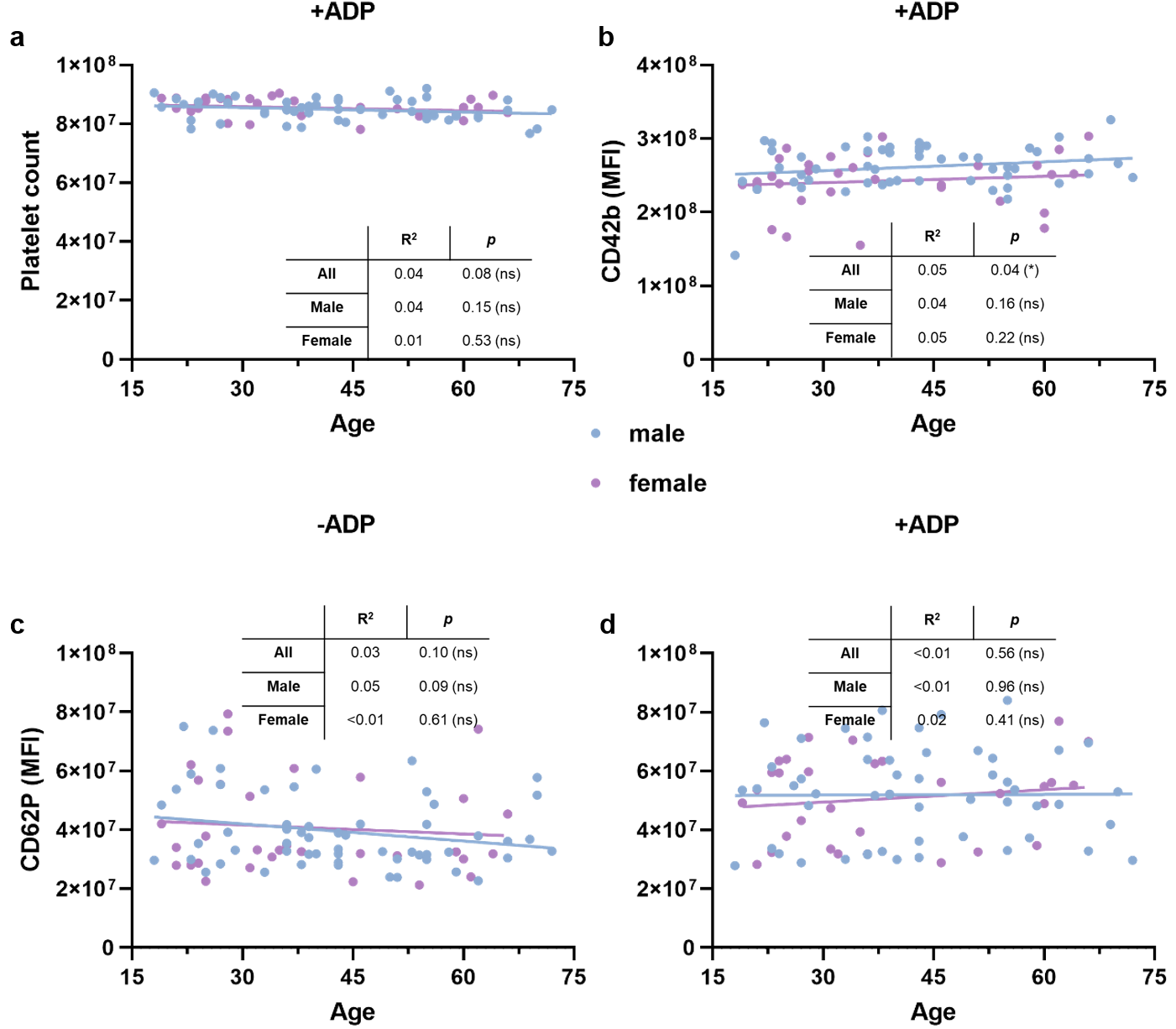


## Figure S7


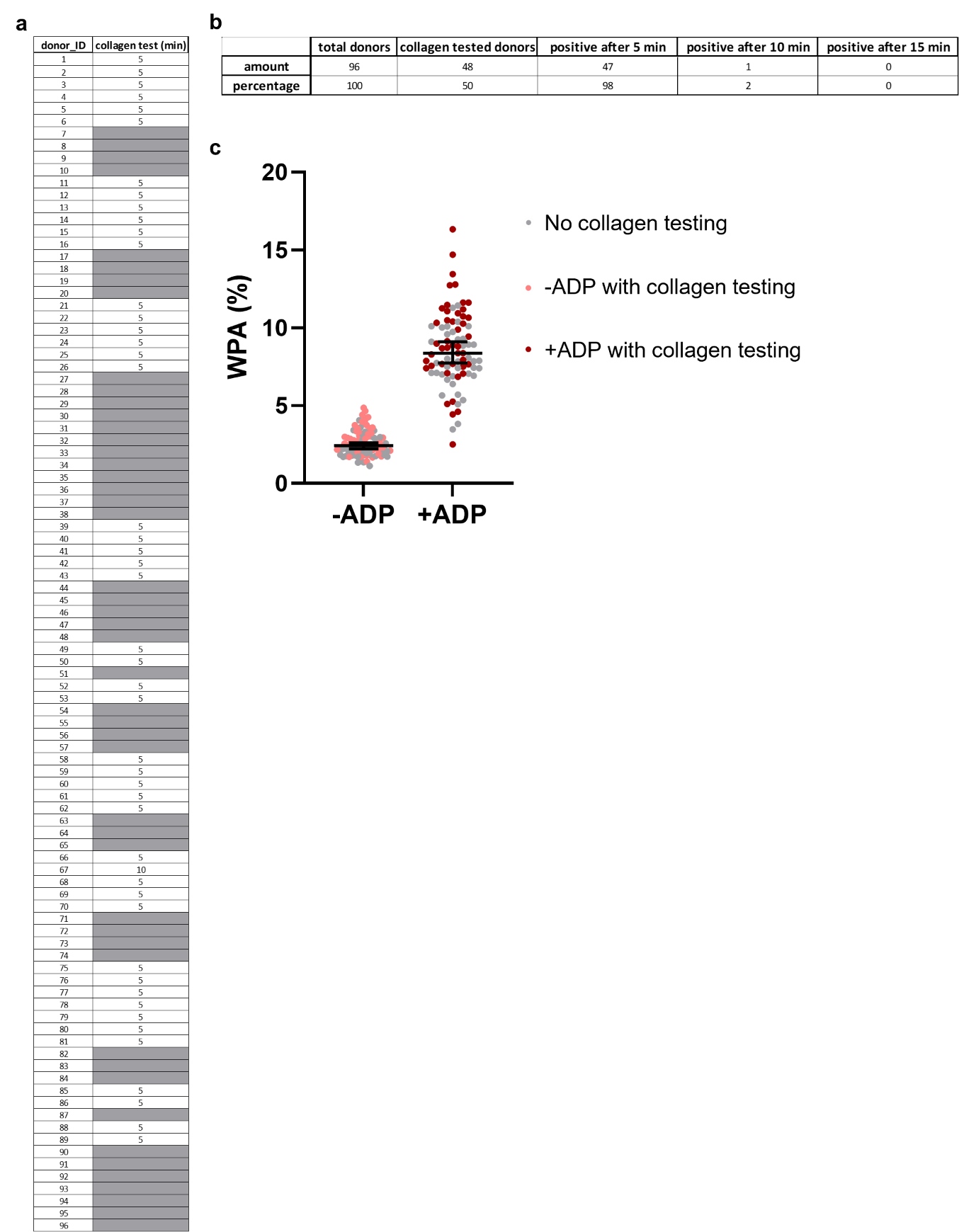

Supplement: Multimedia component 1 [file mmc1.docx]
